# Seven lessons from the coronavirus pandemic for primary health care: A qualitative study of registered and assistant nurses in Sweden

**DOI:** 10.1111/scs.13082

**Published:** 2022-04-24

**Authors:** Per Nilsen, Hanna Fernemark, Ida Seing, Kristina Schildmeijer, Janna Skagerström

**Affiliations:** ^1^ Division of Health and Society Department of Health, Medicine and Caring Sciences Linköping University Linköping Sweden; ^2^ Division of Health and Society Department of Health, Medicine and Caring Sciences Primary Health Care Centre, Lambohov Linköping University Linköping Sweden; ^3^ Department of Behavioral Science and Learning Linköping University Linköping Sweden; ^4^ Department of Health and Caring Sciences Faculty of Health and Life Sciences Linnaeus University Kalmar Sweden; ^5^ Research and Development Unit in Region Östergötland Linköping Sweden

**Keywords:** change, coronavirus, learning, pandemic, primary health care

## Abstract

**Aim:**

The aim of this study was to explore lessons from the pandemic by registered and assistant nurses in Swedish primary health care (PHC) of potential relevance for the future operation of PHC.

**Methods:**

Twenty‐one semi‐structured interviews were conducted with registered and assistant nurses. We used a purposeful sampling strategy to achieve a diverse sample with regard to size and location of PHC centres. Data were analysed using qualitative content analysis.

**Results:**

Analysis yielded two categories: lessons from the pandemic pertaining to PHC personnel and patient behaviours (adaptability of the personnel; importance of hygiene and maintaining physical distance; and importance of being attentive to illness symptoms) and lessons from the pandemic related to primary healthcare work routines (effectiveness of digital job meetings; advantages of digital patient consultations; importance of keeping infectious patients separate from other patients; and the need to allow only pre‐booked patient appointments).

**Conclusions:**

The seven sub‐categories represent seven lessons from the pandemic. The lessons generated both instrumental knowledge, which the nurses could apply in work‐related decisions, and conceptual knowledge which yielded improved understanding of problems and potential solutions for PHC.

## INTRODUCTION

The spread of coronavirus disease (COVID‐19) has had an impact on societies worldwide, with particularly negative effects on healthcare systems, including increased demand for testing, tracking and tracing capacities, the use of personal protective equipment and scaling‐up of hospital and workforce capacities to manage surges in care demand and overcrowded intensive care units [1, 2]. Primary health care (PHC) has managed a large share of COVID‐19‐related care [3, 4] and has had an important role by reinforcing public health messages, differentiating patients with respiratory symptoms from those with COVID‐19, making an early diagnosis, helping vulnerable people cope with their anxiety about the virus and reducing the demand for hospital services [5, 6].

Primary health care nurses in Sweden and internationally have had a significant role during the pandemic, including triaging patients and identifying suspected cases of infection, coordinating with other healthcare providers and supplying holistic nursing practices in managing multiple infections simultaneously [7]. PHC nurses are also engaged in the vaccination programmes [8].

The pandemic has required many adjustments in health care [2]. Many traditional ways of organising PHC have changed, and some of these changes could potentially be more effective than previous approaches. Although there have been numerous opinion‐based media reports on healthcare professionals' experiences from the pandemic, we have not identified any studies that have empirically investigated this issue. A recent systematic review noted that most research on nurses' experiences during respiratory pandemics or epidemics has focused on acute care nurses [9]. Therefore, the aim of this study was to explore lessons from the pandemic by registered and assistant nurses in Swedish PHC of potential relevance for the future operation of PHC. The lessons PHC will learn from the pandemic may be crucial for patient care and the development of healthcare systems.

## METHODS

### Study design and setting

We used a qualitative approach based on semi‐structured interviews with registered and assistant nurses. The Swedish healthcare system is divided into 21 regions. All citizens are insured by the state and have equal access to health care. In 2019, there were 1140 PHC centres in Sweden. Registered nurses make up 44% and assistant nurses 16% of the total workforce of 25,000 in PHC [10]. The tasks of registered nurses in Swedish PHC vary depending on the specific workplace, but usually include leading and planning the nursing work, handling medication, testing and examination as well as keeping medical records of patients' conditions. Work also involves telephone counselling and assessment of whether patients should wait, book a physician appointment or seek emergency care. The tasks of assistant nurses in Swedish PHC not only vary between workplaces but also account for their experience. Common tasks include taking samples, measuring blood pressure, attending to wounds, assisting during minor surgery, storage and cleaning. Assistant nurses are usually part of PHC teams together with registered nurses and physicians.

### Recruitment of participants

A purposeful sampling strategy was used to achieve a diverse sample with regard to size (number of listed patients) and location (rural, urban and different regions of Sweden) of PHC centres.

We approached six regions by email to recruit participants, of which four agreed to participate. In two regions, participants received information about the study via the PHC management. In another region, four centres were chosen based on our sampling strategy by the PHC management. In the fourth region, we established contact with a researcher/clinician who disseminated study information to relevant PHC centres. We approached 14 registered nurses, 11 of whom agreed to participate, and we contacted ten assistant nurses all of whom agreed to participate. Table 1 provides data on the participants.

**TABLE 1:** Participant characteristics

Characteristics	Number (%)
Gender	
Female	21 (100)
Profession	
Registered nurse	11 (52)
Assistant nurse	10 (48)
Age	
20–30 years	3 (14)
31–40 years	4 (19)
41–50 years	4 (19)
51–60 years	8 (38)
61–70 years	1 (5)
>70 years	1 (5)
Work experience in the current PHC unit	
0–5 years	14 (67)
6–10 years	4 (19)
11–15 years	1 (5)
>15 years	2 (10)

**FIGURE 1:**
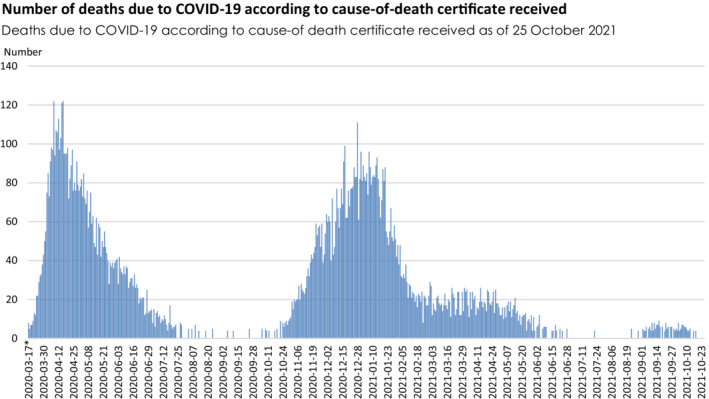
Deaths due to COVID‐19 according to cause‐of‐death certificate received as of 25 October 2021 (*source*: the National Board of Health and Welfare)

The interviews were conducted via Zoom during October–December 2020. This was a period of many workplace restrictions due to the pandemic, making on‐site interviews very difficult. Figure 1 shows the number of deaths due to COVID‐19 (according to cause‐of‐death certificate received), indicating that the interviews were carried out as the second pandemic wave hit Sweden.

### Data collection

The authors developed a semi‐structured interview guide. Lessons from the COVID‐19 pandemic in this study refer to participants' experiences and insights that can be directly linked to the pandemic and may be relevant for the future operations of PHC. The analysis is based on two questions posed to the nurses as part of the interviews: ‘What have you learned from the pandemic?’ and ‘What do you take with you?’ The other interview questions were not used in this study. The interview guide is provided as Supporting Information.

The interviews, lasting between 26 and 48 min, were conducted by HF and KS using Zoom. All interviews were digitally recorded and transcribed verbatim by a professional firm specialised in transcribing interviews. The transcripts were examined for accuracy by the interviewers.

### Data analysis

The data were analysed using qualitative content analysis in accordance with Krippendorff [11], which is a widely used approach deemed appropriate in relation to research question. Content analysis is a technique for analysing texts based on empirical data with an explorative and descriptive character, entailing a structured analysis process to code and categorise the data [11]. The interviews were analysed in several steps. Each author first read all the transcripts to obtain an understanding of the whole. The first author (PN) then reviewed the transcripts and identified coding units in the text that captured various key statements in relation to the study aim. This step involved merging the coding units into context units, which reflected more than one key statement or thought. The context units were then combined by the first author into sub‐categories based on similarity of the content [11]. The sub‐categories were created to be internally homogeneous and externally heterogeneous and were intended to be mutually exclusive [12].

Next, the sub‐categories were merged by the first author under two overarching system levels based on their characteristics. The other authors then discussed the content of the categories and sub‐categories with the first author at several Zoom meetings (the analysis was carried out during a period of on‐site workplace restrictions due to the pandemic) and via emails. These discussions continued until consensus was reached, and no inconsistencies existed. A shared understanding was reached through these discussions to address matters of intersubjectivity and thus strengthen internal validity [11, 13].

Representative quotations from participants were selected by PN, HF and KS, and were discussed with the rest of the team before they were agreed upon. Quotations were then translated from Swedish to English by PN and proposed to the other researchers before final decisions were taken by the whole team. Quotations are marked RN1–11 for registered nurses and AN1–10 for assistant nurses.

### Ethics considerations

The study was approved by the Swedish Ethical Review Authority (2020‐03981 and 2021‐00581) and registered in line with the GDPR. The interviews were conducted from October to December 2020. The interviewed nurses provided informed consent by confirming that they had read and understood the study information concerning the aim of the study, various practical and ethical issues concerning the interview (including data storage) and that they could withdraw participation at any point during the interview.

## RESULTS

**FIGURE 2:**
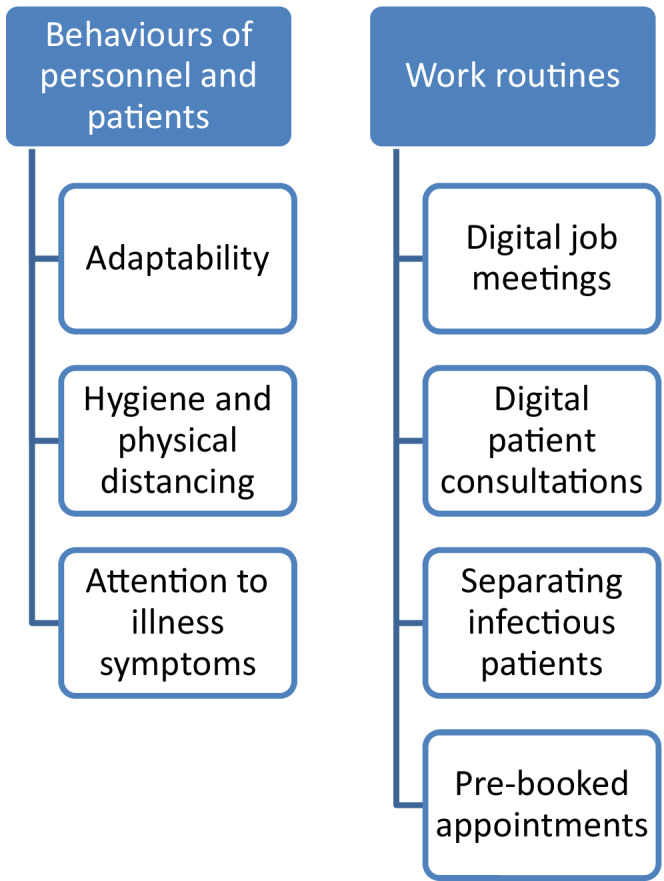
Categories and sub‐categories

Analysis of the interviews yielded two categories: lessons from the COVID‐19 pandemic pertaining to PHC personnel and patient behaviours and lessons from the pandemic related to PHC work routines. The first category consists of three sub‐categories and the second consists of four sub‐categories, providing seven lessons from the pandemic. The categories and sub‐categories are provided in Figure 2.

### Lessons pertaining to personnel and patient behaviour

The participants described three lessons from the COVID‐19 pandemic related to the behaviour of PHC personnel and patients of potential relevance for the future operations of PHC.

The first lesson showed that PHC personnel have considerable adaptability to respond efficiently to the COVID‐19 crisis. Many work routines changed during the pandemic, but the participants were genuinely impressed with the personnel's ability to adapt to the new circumstances.

If one can brag a little, I think we have been fantastic and been flexible. We have made quick changes, changed many times. Everyone has been onboard; we have solved it. And I think it has been fantastic with the whole gang at the health centre. (RN2)

I take with me [from the pandemic] that we, employees and managers, are more flexible than we think. We are more adaptive to change than we think. If you are faced with a fact, you are incredibly flexible. (RN4)

The second lesson from the pandemic concerned the importance of various forms of hygiene and maintaining physical distance at work and in society. The participants noted that authorities' repeated public health messages seemed to have an impact on the hygiene‐related behaviour of the general population as well as within their PHC unit. Participants commented that there was a considerable reduction in the number of patients with influenza, which they attributed to improved hygiene among citizens. They also commented that the pandemic led to increased awareness of and compliance with existing hygiene guidelines.

Hygiene is actually a thing, which I think has helped us, that we have stayed quite healthy actually. You didn't think of it before, even if we are good with washing, disinfection and keeping clean and so on. But it's one thing that I think has helped us here in this workplace. (AN5)

We have hardly seen any patients with streptococcal infection. We have not heard of winter vomiting and stomach diseases. So we have learned that if we wash our hands, we can avoid a lot of these problems. (AN9)

The third lesson from the pandemic was the importance of being more attentive than before to their own symptoms of illness and they were more likely than previously to stay home from work when feeling the slightest bit ill. Participants said they were used to work despite not feeling well because they knew that their absence would increase their colleagues' workload. The pandemic seemed to change the nurses' reasoning and behaviours because the authorities in Sweden kept repeating the message that people must stay home from work when not feeling well to avoid transmitting disease.

Even then, if they [doctors and nurses] have the slightest cold, they should not come to work. If we find that a doctor reports sick in the morning, who may have a fully booked day, then it is up to the staff to reorganize the schedule. (RN1)

It's common among healthcare workers that, “I can have a little runny nose and cough, but I can work anyway.” Just recently I talked to someone who works as an occupational therapist; they visit the old and sick and try out aids. She had a colleague who had cold symptoms but went to work; then he took a COVID‐19 test and he was positive. (RN7)

### Lessons pertaining to changes in primary healthcare work routines

The participants described four lessons from the COVID‐19 pandemic that concerned changes in PHC work routines of potential relevance for the future operations of PHC. These changes were introduced before or during the pandemic and the participants believed they should be made permanent because they could contribute to building an improved PHC.

The fourth lesson from the pandemic concerned recognition of the effectiveness of using digital meetings. Participants argued for a reduction in the number of physical on‐site job meetings in favour of more digital meetings in the future. Although digital meetings were used occasionally before the pandemic, the number of such meetings increased manyfold as they replaced traditional on‐site job meetings during the pandemic. Participants acknowledged that digital meetings saved a great deal of time, particularly for those who had to travel long distances to attend meetings at a more centrally located PHC unit. The participants believed digital job meetings should be utilised to a greater extent in the future.

What you can learn is that you do not have to go to all these meetings. You can of course reduce that. You cannot take blood pressure and listen to heart and lungs through a phone, but you can have these [job] meetings. You will save time if you avoid sitting in the car to travel to meetings. (AN3)

Now we can have meetings via video to reduce driving or travelling in general. We have started with the fact that you can work from home. For example, you can take phone calls from home when someone is home and is ill, so you can continue with that even though there is no pandemic. (AN4)

The fifth lesson from the pandemic concerned the advantages of digital patient consultations, which became much more common during the pandemic. Participants were positive about digital patient interaction because it saved time for both the personnel and patients who did not have to travel to a PHC unit unless it was necessary.

I think we should continue with [digital consultations]. Yes, I believe in it. And there are many patients who are very happy with it, can sit at work and maybe have a doctor's visit during lunch instead of having to go away. It saves time for the patients as well. (AN2)

It's fun, comical how fast the [digital consultations] development has gone. It is now web‐based, a webcam here and Zoom meetings there. And I think that is great and I wonder why it did not start earlier, because there is a lot you can do today in healthcare via the web. (RN9)

For the sixth lesson, participants described the importance of keeping infectious patients physically separate from other patients in PHC. The PHC units involved in the study introduced separate entrances and waiting rooms for potential COVID‐19 patients to reduce the risk of transmitting the virus to others during visits. Participants recognised that this was an advantageous routine that should be sustained.

Infectious patients in an individual room, it's something we have started now and will continue with, because there is always a risk in having them to the waiting room, even if it is not COVID‐19. It could be another cold or something that can infect someone in a risk group. (AN4)

I think that in the long run, we will probably also need to change our waiting rooms. Here, we have 18 doctors who all have a patient at 9 o'clock so the waiting rooms will be very full. We have to solve it practically. (AN6)

The seventh lesson from the pandemic concerned the need to allow only pre‐booked appointments with PHC personnel. They argued for the end of the existing routine of allowing patients to merely show up at a PHC unit to seek care. Participants recognised that the current routine was sub‐optimal in terms of being able to manage the patients who visit the unit, making it difficult to handle patients who might transmit infection and disease. The drop‐in routine also resulted in unpredictability for the PHC personnel because the work burden was difficult to predict and could be very high some days.

During flu vaccination period, we have only had [advance] booking; no open drop‐ins. And it has worked very, very well. There have been no waiting times; there has been no [crowding], maximum three patients in a large waiting room. We will probably not go back to [drop‐in] because it was also stressful when there were many patients in line; they often had to work extra hours. (AN10)

We can learn from the fact that we should not have drop‐ins for the vaccination [for COVID‐19]. I do not think we need to have any more drop‐ins, also from a stress point of view. (RN10)

## DISCUSSION

This study explored lessons from the COVID‐19 pandemic with registered and assistant nurses of potential relevance for the future patient care and operations of PHC in Sweden. Seven lessons emerged, based on the accounts of the nurses who were interviewed. The lessons concerned both changes in the behaviour of personnel and patients, and changes in work routines. The learning involved in these changes (i.e. in behaviours and work routines) can be understood in terms of accumulation of two forms of knowledge: instrumental knowledge, which is knowledge that can be utilised in direct applications [14], for example in work‐related decisions, and conceptual knowledge, which is knowledge in terms of enlightening perceptions [14], for example improved understanding of problems and potential solutions for PHC.

One of the lessons concerned the capacity of the PHC personnel to adapt well to the new situation created by the pandemic. The nurses in our study commented that this flexibility contrasted with the view of health care as resistant to change. The pandemic can be characterised as second‐order change, that is a discontinuous change that typically challenges prevailing norms and values, and requires new ways of doing things. In contrast, first‐order change involves making adjustments to an existing process. The cause of second‐order change is usually some form of crisis or strategic change [15, 16].

Change, in general, can be challenging because it contradicts humans' basic need for a stable environment [17]. Research has shown that organisational changes are often associated with employees' psychological uncertainty about how the changes will affect them [18, 19, 20]. This is even more likely with disruption caused by second‐order change, which may generate a great deal of anxiety, frustration and temporary dysfunction [21]. Despite the challenge of the second‐order change triggered by the pandemic, the nurses in our study expressed generally positive attitudes to the changes in response to the pandemic. The findings can be related to a previous study by the research team behind the study [22], in which three characteristics of successful changes were identified in healthcare organisations: nurses and physicians supported changes that allowed for preparation; featured the personnel's involvement; and were perceived as valuable, including having clear patient benefits. Although many changes in response to the pandemic allowed limited time for preparation, most changes had a patient focus and involved the personnel to a large degree, thus fulfilling two of the three ‘success criteria’.

The pandemic seemed to enhance trust among the PHC staff as they worked together to manage the situation. Social trust has been defined as positive expectations that another party, for example one's colleagues, will act benevolently. Trust therefore involves a willingness to be vulnerable and risk that the other party may not fulfil those expectations [23, 24, 25, 26]. Trust is fostered in ambiguous situations, which may lead to positive or negative personal outcomes, when the trusting individual is dependent on one or more other persons for the determination of that outcome and when there is a degree of confidence in the trusted persons' altruism [27]. Social trust acts as a foundation for cooperation and may be particularly important in complex situations such as during a pandemic when information is constantly changing, and knowledge might be lacking [28].

Four of the lessons concerned health‐protective behaviours and routines to avoid transmission of disease: (1) the importance of hygiene and social distancing; (2) being attentive to illness symptoms; (3) separating infectious patients from other PHC patients; and (4) cancelling drop‐in appointments. The participants believed the pandemic improved hygiene in the population, which led to lower rates of patients with influenza in health care. Statistics support their impression as the incidence of seasonal influenza and Calicivirus in Sweden was lower than expected in 2020–2021 [29].

The finding concerning the nurses' willingness to stay home from work when feeling ill contrasts with the self‐sacrificing culture of the nursing profession. Presenteeism attributed to sickness (attending work when ill) and/or job stress (when work stress affects performance) in nursing is more common than in other job sectors [30]. Presenteeism has been found to be costly and linked to many negative outcomes for both nurses and patients [31, 32]. Some of the nurses in our study seemed relieved that the pandemic made it socially acceptable for them to stay home from work when they felt ill. Thus, the second‐order change in the pandemic was strong enough to challenge the prevailing self‐sacrificing culture, enhancing social norms that prioritised health‐protective behaviours.

The nurses favoured pre‐booked appointments because it enabled better surveillance of patients and made it easier to avoid transmission of infection and disease. A previous study by the research team behind this study found that PHC physicians' job satisfaction was negatively influenced by difficulties they perceived with regard to planning work ahead and being prepared for unexpected events [33]. We have not found any studies on nurses that have investigated this particular topic.

Two of the lessons were associated with digitalisation, in terms of job meetings and patient consultations. Research is limited concerning digital job meetings with colleagues in health care. However, a literature review [34] of 73 studies concerning the work environment in various forms of digital telework documented positive aspects for individuals such as improvements with regard to work‐life balance, flexibility, autonomy and control in work. Negative aspects included increased number of working hours and workload, blurred divisions between work and non‐work domains and reduced social support from managers and co‐workers [34]. Studies on digital patient consultations with healthcare professionals, often referred to as telemedicine, show similar results with regard to working conditions [33, 35].

Digital patient consultations increased significantly in Sweden during 2020, not only mirroring the reduced number of physical patient visits to PHC but also reflecting a trend towards greater use of this service. Many of the country's 21 regions more than doubled the number of telemedicine contacts from 2019 to 2020 [36]. Other countries have also seen increased use of telemedicine in response to the pandemic [37, 38].

The pandemic has accelerated the pace of telemedicine in PHC [39]. It seems unlikely that digitalisation rates will fall back to the pre‐pandemic levels, because this way of interacting with patients has been favourably received by both patients and PHC personnel [33]. However, Koivunen and Saranto [40] noted in their review of studies conducted between 1998 and 2015 that insufficient skills and attitudes were perceived to be preventive factors for many nurses. Studies have documented that nurses worry that digital patient consultations will create more work and feelings of loss of visibility and control [41, 42, 43, 44].

Some methodological considerations should be acknowledged when interpreting the findings. Participation in the study was voluntary, that is the nurses who were interviewed may have been particularly interested in the topic although it is difficult to determine how this might have affected the results. The transferability of the results is limited to PHC settings in Sweden.

There are also strengths. The multidisciplinary research team enhanced the credibility of the study because they facilitated different perspectives on the topic. A relatively high number of nurses were interviewed for the study, which enabled us to use quotations from 13 participants, thus enhancing the study's trustworthiness and transparency. Another strength was the fact that the participants came from different geographic regions of Sweden and from both public and private healthcare organisations.

In conclusion, the COVID‐19 pandemic yielded lessons in terms of changes in the behaviour of personnel and patients, and changes in work routines in PHC in Sweden. Registered and assistant nurses gained both instrumental knowledge, which they could apply in work‐related decisions, and conceptual knowledge, which yielded improved understanding of problems and potential solutions for PHC.

In terms of practical implications, it is too early to speculate on the extent to which various changes will become permanent features of future PHC operations. The COVID‐19 pandemic represents a disruptive second‐order change, which is usually viewed as being an irreversible process [15, 16]. Further research is needed to investigate how the pandemic will affect PHC in the longer term. Learning from the COVID‐19 pandemic is crucial because this will not be the last crisis faced by humankind.

## CONFLICT OF INTEREST

The authors report no conflicts of interest in connection with this article.

## AUTHOR CONTRIBUTIONS

PN conceptualised the study. All aspects were discussed with HF, IS, KS and JS. PN drafted the first version of the manuscript with assistance from HF. Later versions of the manuscript were discussed with all the authors as PN continuously revised the manuscript. All the authors approved the final manuscript.

## ETHICAL APPROVAL

The study was approved by the Swedish Ethical Review Authority (no. 2020‐03981).

## Supporting information


**Appendix S1.** Supplementary Information

## References

[1] Primahendra R , Sumbogo TA , Lensun RA , Purwanto A . Handling corona virus pandemic in the Indonesian political context: a grounded theory study. Eur J Mol Clin Med. 2020;7(8):113–29.

[2] Shanafelt T , Ripp J , Trockel M . Understanding and addressing sources of anxiety among health care professionals during the COVID‐19 pandemic. JAMA. 2020;323:2133–4. 10.1001/jama.2020.5893 32259193

[3] Mash B . Primary care management of the coronavirus (COVID‐19). S Afr Fam Pract. 2020;62:a5115. 10.4102/safp.v62i1.5115 PMC7577343 32242438

[4] Rawaf S , Allen LN , Stigler FL , Kringos D , Yamamoto HQ , van Weel C , et al. Lessons on the COVID‐19 pandemic, for and by primary care professionals worldwide. Eur J Gen Pract. 2020;26:129–33. 10.1080/13814788.2020.1820479 32985278 PMC7534357

[5] Krist AH , DeVoe JE , Cheng A , Ehrlich T , Jones SM . Redesigning primary care to address the COVID‐19 pandemic in the midst of the pandemic. Ann Fam Med. 2020;18:349–54. 10.1370/afm.2557 32661037 PMC7358035

[6] World Health Organization . Role of primary care in the COVID‐19 response; 2020. https://apps.who.int/iris/bitstream/handle/10665/331921/Primary‐care‐COVID‐19‐eng.pdf?sequence=1&isAllowed=y. Accessed 5 May 2021.

[7] Xie J , Tong Z , Guan X , Du B , Qiu H , Slutsky AS . Critical care crisis and some recommendations during the COVID‐19 epidemic in China. Intensive Care Med. 2020;46:837–40. 10.1007/s00134-020-05979-7 32123994 PMC7080165

[8] Alexander D. Symposium explores the role of nurses in vaccine rollout. Johns Hopkins University Hub; 2021. https://hub.jhu.edu/2021/03/02/mission‐possible‐symposium/. Accessed 7 April 2021.

[9] Fernandez R , Lord H , Halcomb E , Moxham L , Middleton R , Alananzehm L , et al. Implications for COVID‐19: a systematic review of nurses' experiences of working in acute care hospital settings during a respiratory pandemic. Int J Nurs Stud. 2020;111:103637. 10.1016/j.ijnurstu.2020.103637 32919358 PMC7206441

[10] SKR . Regionanställd personal 2019 [Regionally employed staff 2019]; 2020. skr.se/arbetsgivarekollektivavtal/uppfoljninganalys/personalstatistik/personalenisiffror/tabellerregionanstalldpersonal2019.32644.html. Accessed 5 April 2021 (in Swedish).

[11] Krippendorff K. Content analysis: an introduction to its methodology. 2nd ed. Thousand Oaks, CA: Sage; 2004.

[12] Schreier M . Qualitative content analysis. In: Flick U , editor. The sage handbook of qualitative analysis. Los Angeles, CA: Sage; 2013. p. 170–83.

[13] Patton MQ . Qualitative research & evaluation methods. 3rd ed. Thousand Oaks, CA: Sage; 2002.

[14] Nutley SM , Walter I , Davies HTO . Using evidence. Bristol: The Policy Press; 2007.

[15] Pennington G . Guidelines for promoting and facilitating change. New York: LTSN Generic Centre; 2003.

[16] Weick KE , Quinn RE . Organisational change and development. Annu Rev Psychol. 1999;50:361–86. 10.1146/annurev.psych.50.1.361 15012461

[17] Grama B , Todericiu R . Change, resistance to change and organizational cynicism. Stud Bus Econ. 2016;11(3):47–54. 10.1515/sbe-2016-0034

[18] Bernerth J , Walker HJ , Harris SG . Change fatigue: development and initial validation of a new measure. Work Stress. 2011;25(4):321–37. 10.1080/02678373.2011.634280

[19] Ead H . Change fatigue in health care professionals‐‐‐‐an issue of workload or human factors engineering? J Perianesth Nurs. 2015;30(6):504–15. 10.1016/j.jopan.2014.02.007 26596386

[20] Rafferty AE , Griffin MA . Perceptions of organizational change: a stress and coping perspective. J Appl Psychol. 2006;91:1154–62. 10.1037/0021-9010.91.5.1154 16953776

[21] Watkins DM . Leadership transitions: the Watkins collection. 3rd ed. Brighton, MA: Harvard Business Review Press; 2009.

[22] Nilsen P , Seing I , Ericsson C , Birken SA , Schildmeijer K . Characteristics of successful changes in health care organizations: an interview study with physicians, registered nurses and assistant nurses. BMC Health Serv. 2020;20:147. 10.1186/s12913-020-4999-8 PMC7045403 32106847

[23] van Dijke M , De Cremer D , Mayer DM . The role of authority power in explaining procedural fairness effects. J Appl Psychol. 2010;95(3):488–502. 20476828 10.1037/a0018921

[24] Edwards JR , Cable DM . The value of value congruence. J Appl Psychol. 2009;94(3):654–77. 19450005 10.1037/a0014891

[25] Lane PJ , Salk JE , Lyles MA . Absorptive capacity, learning, and performance in international joint ventures. Strateg Manag J. 2001;22(12):1139–61.

[26] Selnes F . Antecedents and consequences of trust and satisfaction in buyer‐seller relationships. Eur J Mark. 1998;32(3/4):305–22.

[27] Frost T , Stimpson DV , Maughan MRC . Some correlates of trust. J Psychol. 1978;99(1):103–8. 650598 10.1080/00223980.1978.9921447

[28] Dohle S , Wingen T , Schreiber M . Acceptance and adoption of protective measures during the COVID‐19 pandemic: the role of trust in politics and trust in science. Soc Psychol Bull. 2020;15(4):1–23.

[29] Folkhälsomyndigheten . Calicivirusrapporter säsong 2020/2021 [Calicivirus reports season 2020/2021]; 2021. https://www.folkhalsomyndigheten.se/folkhalsorapportering‐statistik/statistik‐a‐o/sjukdomsstatistik/calicivirus‐veckorapporter/calicivirusrapporter‐sasong‐2020‐2021/. Accessed 5 April 2021.

[30] Aronsson G , Gustafsson K , Dallner M . Sick but yet at work. An empirical study of sickness presenteeism. J Epidemiol Community Health. 2000;54(7):502–9. 10.1136/jech.54.7.502 10846192 PMC1731716

[31] Bergström G , Bodin L , Hagberg J , Lindh T , Aronsson G , Josephson M . Does sickness presenteeism have an impact on future general health? Int Arch Occup Environ Health. 2009;82(10):1179–90. 10.1007/s00420-009-0433-6 19504117

[32] Pilette PC . Presenteeism in nursing: a clear and present danger to productivity. J Nurs Admin. 2005;35:300–3. 10.1097/00005110-200506000-00006 15951705

[33] Fernemark H , Skagerström J , Seing I , Ericsson C , Nilsen P . Digital consultations in Swedish primary health care: a qualitative study of physicians' job control, demand and support. BMC Fam Pract. 2020;21:241. 10.1186/s12875-020-01321-8 33234111 PMC7684852

[34] Eklund J , Palm K , Bergman A , Rosenblad C , Aronsson G . Work environment of the future – trends, digitalization and employment forms: three systematic reviews. Report 2020:1. Swedish Agency for Work Environment Expertise. https://media.sawee.se/2020/06/Work‐environment‐of‐the‐future‐%E2%80%93‐trends‐digitalization‐and‐employment‐forms.pdf. Accessed 12 February 2022.

[35] Kaminsky E , Röing M , Björkman A , Holmström IK . Telephone nursing in Sweden: a narrative literature review. Nurs Health Sci. 2017;19:278–86. 10.1111/nhs.12349 28618087

[36] Cederberg J . Flerfaldig ökning av digital vård [Multiple increase in digital care]. Läkartidningen. 2021;13–4. (in Swedish).

[37] Alexander GC , Tajanlangit M , Heyward J , Mansour O , Qato DM , Stafford RS . Use and content of primary care office‐based vs telemedicine care visits during the COVID‐19 pandemic in the US. JAMA Netw Open. 2020;3(10):e2021476. 10.1001/jamanetworkopen.2020.21476 33006622 PMC7532385

[38] Sigurdsson EL , Blondal AB , Jonsson JS , Tomasdottir MO , Hrafnkelsson H , Linnet K , Sigurdsson JA . How primary healthcare in Iceland swiftly changed its strategy in response to the COVID‐19 pandemic. BMJ Open. 2020;10:e043151. 10.1136/bmjopen‐2020‐043151 10.1136/bmjopen-2020-043151 PMC7722808 33293329

[39] Horgan D , Hackett J , Westphalen CB , Kalra D , Richer E , Romao M , et al. Digitalisation and COVID‐19: the perfect storm. Biomed Hub. 2020;5:1341–63. 10.1159/000511232 33564668 PMC7573902

[40] Koivunen M , Saranto K . Nursing professionals' experiences of the facilitators and barriers to the use of telehealth applications: a systematic review of qualitative studies. Caring Sci. 2018;32:24–44. 10.1111/scs.12445 28771752

[41] Barbosa SFF , Abbott P , Dal Sasso GTM . Nursing in the digital health era. J Nurs Scholarsh. 2021;53(1):5–6. 10.1111/jnu.12620 33410221

[42] Öberg U , Orre C‐J , Isaksson U , Schimmer R , Larsson H , Hörnsten Å . Swedish primary healthcare nurses' perceptions of using digital eHealth services in support of patient self‐management. Scand J Caring Sci. 2018;32:961–70. 10.1111/scs.12534 28960451

[43] Porter‐O'Grady T . Turning the page: nursing in the digital age and beyond. Nurs Manag. 2019;50(9):40–7. 10.1097/01 31460896

[44] Wilson R . Digital technology: why nurses need to be at the Centre of new developments. Nurse Res. 2020;28(2):6–8. 10.7748/nr.28.2.6.s2

