# Supplementary material for: Seven lessons from the coronavirus pandemic for primary health care: A qualitative study of registered and assistant nurses in Sweden
Source: Scand J Caring Sci. 2022 Apr 24:10.1111/scs.13082. Online ahead of print. doi: 10.1111/scs.13082 (PMC9115448; doi:10.1111/scs.13082)
Supplement: Supplementary file 1 — Appendix S1. Supplementary Information [file SCS-9999-0-s001.docx]

Yellow-marked questions = analyzed in the study

**SWEDISH: Frågeguide**

Muntlig introduktion

(Presentation av aktuell intervjuande forskare, namn, titel, arbetsplats)

Projektet handlar om de stora förändringar som Coronapandemin inneburit och innebär för primärvården och dess medarbetare i Sverige. Syftet är att undersöka hur medarbetare i primärvården upplevt arbetsmiljön under och (förhoppningsvis) efter pandemin, med fokus på upplevd stress, förändringar i fysisk och psykosocial arbetsmiljö samt eventuella fördelar och nackdelar för vården som pandemin orsakat. Fokus för intervjuerna kommer ligga på dina upplevelser och tankar kring dessa förändringar.

Frågor

Personliga erfarenheter på arbetsplatsen

Kan du berätta, lite kortfattat, om din arbetsplats?

(Patientklientel, landsbygd/stad, antal anställda, coronatyngd/hur drabbad enheten varit av corona?)

Kan du beskriva vilka förändringar som gjorts på din arbetsplats i och med Covid-19?

(Nya arbetssätt/digitalisering, organisatoriska förändringar, nya arbetsuppgifter, administration ökad/minskad, personalmöten)

Vilka förändringar har påverkat dig mest?

Hur skulle du beskriva förändringarnas genomförande/implementering?

(Delaktighet, tidsperspektiv/framförhållning, upplevd nytta? Bottom-up? Top-down?)

Kan du beskriva hur dessa förändringar påverkat din arbetsmiljö?

(Arbetsmiljö, stress, oro, ändrade arbetsuppgifter, nya prioriteringar, ändrade arbetstider, arbetsbörda)

Har relationen med patienter påverkats och i så fall hur?

(Ändrade sätt att träffa patienter? Ökad digital mottagning? Involvering av anhörig, t.ex. vid kognitiv svikt hos patient, vård i livets slutskede?)

Har de sociala relationerna på arbetsplatsen förändrats i och med pandemin? Hur?

(Kontakter mellan vårdgivare/slutenvård/remissinstanser, samarbete inom arbetsplatsen)

Har du någon gång under arbetet under Coronapandemin känt oro för din egen hälsa?

Beskriv gärna hur och varför.

(Stress, oro för egen smitta)

Vad kan vi lära oss av Coronapandemin? Vad tar du med dig?

(Organisatoriskt, personligen, kollegialt på arbetsplatsen och mellan andra kliniker, patientrelation, digitala vårdmöten?)

Är det något ytterligare du vill tillägga?

Några avslutande personliga frågor.

Demografiska data:

Ålder

Kön

Antal år i yrket?

Antal år på aktuell arbetsplats?

Vi planerar för uppföljande intervjuer om ca 2 år, kan du tänka dig att delta då igen? Får vi spara dina kontaktuppgifter för detta ändamål?

Tack för din medverkan!

**ENGLISH: Interview guide** (translated from Swedish)

Oral introduction

(Presentation of current interviewing researcher, name, title, workplace)

The project concerns changes that the Corona pandemic has entailed and what they mean for primary care and its employees in Sweden. The purpose is to investigate how employees in primary care experienced the work environment during and (hopefully) after the pandemic, with a focus on perceived stress, changes in the physical and psychosocial work environment and any benefits and disadvantages for the care caused by the pandemic. The focus of the interviews will be on your experiences and thoughts about these changes.

Questions

Personal experiences in the workplace

Can you tell us a little bit about your workplace?

(Patient clientele, rural or city location, number of employees, how affected has the unit been by corona?)

Can you describe what changes have been made to your workplace due to COVID-19?

(New ways of working / digitization, organizational changes, new tasks, administration increased/decreased, staff meetings)

What changes have affected you the most?

How would you describe the implementation of the changes?

(Participation, time perspective, planning/foresight, perceived benefits, bottom-up/top-down)

Can you describe how these changes have affected your work environment?

(Work environment, stress, anxiety, changed work tasks, new priorities, changed working hours, workload)

Has the relationship with patients been affected and if so, how?

(Changed ways of meeting patients? Increased digital reception? Involvement of relatives, e.g. in cognitive impairment in patients, end-of-life care?)

Have the social relations in the workplace changed with the pandemic? How?

(Contacts between care providers, inpatient care, referral bodies, collaboration within the workplace)

Have you ever felt worried about your own health while working during the Corona pandemic? Feel free to describe how and why.

(Stress, anxiety about own infection)

What can we learn from the Corona Pandemic? What do you take with you?

(Organizational, personal, collegial in the workplace and between other clinics, patient relationship, digital care meetings)

Is there anything else you want to add?

Some concluding personal questions.

Demographic data:

Age

Sex

Number of years in the profession?

Number of years in the current workplace?

We are planning for follow-up interviews in about 2 years, would you consider participating then again? May we save your contact information for this purpose?

Thank you for your participation!
